# Zika: An emerging disease requiring prevention and awareness

**DOI:** 10.1371/journal.pntd.0006486

**Published:** 2018-06-21

**Authors:** Rebekah Elliott, Tuhina Banerjee, Santimukul Santra

**Affiliations:** Department of Chemistry, Pittsburg State University, Pittsburg, Kansas, United States of America; Fundacao Oswaldo Cruz, BRAZIL

## Global emergence of the Zika virus and efforts to contain the geographic expansion

The Zika virus (ZIKV) was overlooked as a potential threat for more than half a century. It was discovered first in a Rhesus monkey in 1947 in the Zika Forest of Uganda, hence its name, but not until its emergence in 2007 did the flavivirus grab the attention of researchers [1]. Originally, it was thought that the infection was isolated to only sporadic appearances in Asia and Africa, but this idea quickly changed with the first infections documented outside of its regional endemicity [1]. With an estimated three-fourths of the residents of the Confederated States of Micronesia in Yap Island affected [2], ZIKV went from a tropical infection to a potential health risk. The virus, however, classified with other flaviviruses, such as dengue (DENV), yellow fever (YFV), and chikungunya (CHIKV), seemed to be relatively harmless, being self-limiting and presenting with only mild symptoms similar to influenza [2]. Studies documented that around 80% of those infected were asymptomatic, and in fact, most infected today may not even know they have it [3]. However, since then, ZIKV has transitioned from a minor concern to a global health emergency [4]. Not only has it spread rapidly to other countries, but there are more data evidencing causal relationships to neurological disorders in unborn children with infected mothers, as well as more recent evidence supporting a link between ZIKV and neurological disorders in adults [5]. Because it is mainly asymptomatic and because of the grave consequences this can have on developing fetuses, the virus has come under global investigation, with the World Health Organization (WHO) officially declaring ZIKV a Public Health Emergency of International Concern (PHEIC) in 2016 [6]. Prior to 2014, there were no documented ZIKV infections in the Americas, but by 2016, there were 170,000 confirmed cases and 515,000 suspected ones, although numbers as high as 1.9 million suspected cases have been estimated for the same time period [7, 8].

Pathogenicity on the population level is being analyzed to understand, prevent, and treat this emerging disease. By October 2016, ZIKV outbreaks were being reported in more than 60 countries, with 1 million cases alone in Brazil [4]. These figures continued to increase at an alarming rate, with estimates ranging from 35 countries [3] to as many as 84 countries from the beginning of 2016 to 2017 [9]. Of great concern is the evidence accruing validating the hypothesis that ZIKV is causally linked to microcephaly and other neurological disorders in newborns, fetal malformation [4], and now Guillain-Barré syndrome (GBS) in adults [5]. According to WHO, by March 2017, 23 countries or territories had reported an increase in GBS cases, suggesting a causal link to ZIKV, and 31 had reported increases in central nervous system malformations, with preliminary evidence associating this with ZIKV infection [9]. The virus, spread commonly through the mosquito vector *Aedes aegypti*, also can be transmitted through sexual contact as well as through blood transfusions [3, 4]. The arbovirus is also thought to be potentially transmitted through saliva; preliminary studies, however, suggest transmission through saliva is low [10].

## Combatting the disease with different diagnostic methods

Ways to combat the outbreak include practical considerations. One of the main emphases needs to be low-cost and rapid field detection, especially in low-resource settings where assays such as real-time reverse transcription polymerase chain reaction (RT-PCR) require specialized laboratories and skilled professionals [11]. RT-PCRs have been considered the accepted standard for viral molecular detection due to high selectivity and fairly high sensitivity, but PCR platforms require multi-temperature heating of samples for denaturation, annealing, and extension [11], as well as an additional incubation step for a total reaction time of 2 hours [8]. However, costs and time considerations are not the only complications facing accurate and rapid testing. Cross-reaction of previously infected individuals with other flaviviruses, such as DENV and CHIKV [12], has also proven problematic leading to misdiagnoses, especially with similar clinical presentations and similar areas of endemicity [8].

Isothermic nucleic acid amplification tests (NAATs) show promise, proving successful even with low loads of ZIKV. This is important as ZIKV RNA is frequently not detectable in the blood until several days after symptom manifestation, with one recent study finding the mean time for viremia detection to be 10.4 days, with a range from 0 to 53 days [12]. A new assay, the reverse transcription strand invasion-based amplification (RT-SIBA), showed positive results within 18 to 22 minutes for all 3 ZIKV strains and no cross-reaction with other flaviviruses [13]. This is one of many isothermal NAATs being developed for accuracy, portability for field testing, and low cost, including loop-mediated isothermal amplification (LAMP), helicase-dependent amplification (HAD), strand displacement amplification (SDA), and more [11].

With the currently recommended RT-PCR for detecting ZIKV being limited and despite new assays and detection methods being discovered, biosensors and nanotechnology may become the new standard. Biosensors are selective for a specific analyte, even in a sample containing contaminants [14]. This could be useful in the future in circumventing cross-reaction with other antigens in testing, as in the case with ZIKV, DENV, and CHIKV, if they could be programed to detect specific virus antigens. Their use is already pervasive in the medical field and continually being refined. Self-assembly techniques along with portability and submicron dimensions means biosensors could potentially be significant in designing field assays, allowing for more sensitive detection of ZIKV and other viruses.

Nanotechnology may already be providing some insights into the cross-reaction of antibodies in flaviviruses. One study utilized the antibody to antigen relationship, focusing specifically on an anti-Zika IgG monoclonal antibody (Z-ab) by using functionalized nanoparticles (MRNPs) with Z-ab [15]. The study indicates that there is multiple receptor binding between the full-length ZIKV envelope protein (ZENV) with AXL, HSP70, and TIM-1 [15]. This is potentially significant in understanding the cross-reactivity of ZIKV, given that AXL is a well-known receptor for flaviviruses [16], HSP70 has been specifically linked to the flavivirus causing neurotropism in Japanese encephalitis [17], and TIM-1 has been shown to be a receptor for both DENV and Ebola [15].

## Preventing the rapid spread

On the molecular level, understanding the virus—including structure, replication, and transmission—is essential. New research points to the binding and entry stage as vital to ZIKV propagation [15]. In the study conducted by Shelby and colleagues, favored binding sites for ZIKV were indicated using an innovative magnetic relaxation technology. Using a nanoplatform inhibitor, researchers were then able to test small molecules for inhibitor activity with promising results, including crizotinib, which was shown to decrease Z-ab binding with AXL-receptors [15]. In another study examining inhibitory effects on the propagation of ZIKV at the entry of the virus to the host cell, inhibition effects were seen with polyphenol (-)-epigallocatechin gallate (EGCG), found in large numbers in green tea [18].

Vaccine development currently underway focuses on neutralizing antibodies, utilizing nucleic acid vaccines, inactivated virions, and live-attenuated ZIKV in order to stimulate neutralizing antibodies against proteins present on the surface of the virion [2]. Some of the most recent progress in understanding the flavivirus and immune response has been discovering that activation of a specific killer cell immunoglobulin-like receptor (KIR2DS2) targets short but highly conserved viral peptides of the flavivirus genus [19]. There is also some speculation on vaccine development, utilizing antibodies found in previously infected individuals with DENV, that have shown ZIKV-neutralizing antibodies [20]. According to the WHO vaccine pipeline tracker, there are currently 12 active vaccine candidates in trial, with 2 of those having proceeded to phase II. While the list has reduced from 45 candidates in March 2017, several are nearing anticipated completion dates in 2018, barring setbacks [21].

## Precautions and awareness

While there is no current vaccine or treatment, researchers are making advancements in understanding the virus by studying transmission and molecular structure of the virus to impede the progression of this global health threat. With numbers continuously changing as to how many are infected and new research data covering varied aspects of molecular structure, diagnostic tools, binding properties, and more, it is hard to stay abreast of current research. The unchanging factor is that resources and research are needed to learn about the virus and how to control the spread and effects of this disease.

In the meantime, the top priority in prevention needs to be protection and/or testing for sexually active women of child-bearing age that could become or are infected. Testing for the virus could also prove beneficial for women living in endemic or infected areas that are considering having children. Men and women both should be aware of possible infection, however, as more evidence emerges that ZIKV is able to be transmitted sexually. Precautions when traveling or if living in an infected region are important, with mosquito repellent being a basic but effective means of prevention. The main precaution is limiting mosquito bites by means of pesticides or exposure, with public awareness in infected regions essential. Taking measures to either avoid times outdoors when mosquitoes are active, such as dusk, and/or wear protective clothing or repellent is prudent. Using screens for open windows and doors is also a practical prevention policy.
